# Double-Negative T (DNT) Cells in Patients with Systemic Lupus Erythematosus

**DOI:** 10.3390/biomedicines12010166

**Published:** 2024-01-12

**Authors:** Dimitri Poddighe, Kuanysh Dossybayeva, Samat Kozhakhmetov, Rafail Rozenson, Maykesh Assylbekova

**Affiliations:** 1 School of Medicine, Nazarbayev University, Astana 010000, Kazakhstan; kuanysh.dossybayeva@nu.edu.kz; 2 Clinical Academic Department of Pediatrics, National Research Center for Maternal and Child Health, University Medical Center, Astana 010000, Kazakhstan; maykesh.asylbekova@umc.org.kz; 3 Center for Life Science, National Laboratory Astana, Astana 010000, Kazakhstan; skozhakhmetov@nu.edu.kz; 4 Department of Children’s Diseases n.1, Astana Medical University, Astana 010000, Kazakhstan; rozensonrafail@yandex.ru

**Keywords:** double-negative T cells, DN T cells, DNT cells, systemic lupus erythematosus, lupus, MRL/lpr mouse

## Abstract

Double-negative T (DNT) cells are a rare and unconventional T-lymphocyte subpopulation lacking both CD4 and CD8 markers. Their immunopathological roles and clinical relevance have yet to be elucidated. Beyond autoimmune lymphoproliferative syndrome (ALPS), these cells may also play a role in rheumatic disorders, including systemic lupus erythematosus (SLE); indeed, these two diseases share several autoimmune manifestations (including nephritis). Moreover, one of the main experimental murine models used to investigate lupus, namely the MRL/lpr mouse, is characterized by an expansion of DNT cells, which can support the production of pathogenic autoantibodies and/or modulate the immune response in this context. However, lupus murine models are not completely consistent with their human SLE counterpart, of course. In this mini review, we summarize and analyze the most relevant clinical studies investigating the DNT cell population in SLE patients. Overall, based on the present literature review and analysis, DNT cell homeostasis seems to be altered in patients with SLE. Indeed, most of the available clinical studies (which include both adults and children) reported an increased DNT cell percentage in SLE patients, especially during the active phases, even though no clear correlation with disease activity and/or inflammatory parameters has been clearly established. Well-designed, standardized, and longitudinal clinical studies focused on DNT cell population are needed, in order to further elucidate the actual contribution of these cells in SLE pathogenesis and their interactions with other immune cells (also implicated and/or altered in SLE, such as basophils), and clarify whether their expansion and/or immunophenotypic aspects may have any immunopathological relevance (and, then, represent potential disease markers and, in perspective, even therapeutic targets) or are just an unspecific epiphenomenon of autoimmunity.

## 1. Introduction

Double-negative T (DNT) cells are a rare T-lymphocyte subpopulation lacking both CD4 and CD8 markers; however, they express the αβ or γδ T-cell receptor (TCR). Recent evidence suggested that DNT cells can be generated through both thymus-dependent (by escaping from the negative selection process) and thymus-independent (probably from activated peripheral lymphocytes that, under specific circumstances, lose the expression of their CD4 or CD8 markers) pathways, even though the exact ontogeny process has yet to be fully elucidated [1]. Recently, some evidence suggested that DNT cells could derive from autoreactive CD8^+^ T cells, especially in the context of autoimmunity [2,3]. Different mechanisms may be implicated in the generation of DNT cells expressing TCRαβ or TCRγδ, which are supposed to have different functional and phenotypic characteristics [1]. According to some recent evidence, mainly derived from experimental models, DNT cells can display both inflammatory and immunoregulatory (anti-inflammatory or suppressive) functions [4].

In general, compared to TCRγδ^+^, TCRαβ^+^ DNT lymphocytes have been more extensively investigated so far and, indeed, most studies specifically refer to them as DNT cells. Indeed, these (TCRαβ^+^) DNT cells initially attracted medical interest since their expansion represents a specific hallmark of the autoimmune lymphoproliferative syndrome (ALPS), wherein (TCRαβ^+^) DNT cell count >1.5% of total lymphocytes and/or >2.5% of CD3^+^ lymphocytes represent one of the diagnostic criteria [5,6]. Due to the coexistence of several and different types of autoimmune manifestations in ALPS patients, these DNT cells have also been studied in the context of rheumatic disorders and especially in systemic lupus erythematosus (SLE). Indeed, SLE shares with ALPS an important clinical heterogeneity, since almost every organ or system can be potentially affected by the immunopathological process [7,8].

SLE is an autoimmune disease with a very variable clinical expression: skin, musculoskeletal, hematological, and renal disorders are the most frequent manifestations, but all organs can be virtually targeted by the underlying immunopathological process, as already mentioned. Among them, (lupus) nephritis is the most relevant complication from a prognostic point of view. Such a protean clinical picture is also associated with a large and variable production of autoantibodies; however, double-stranded DNA (anti-dsDNA) antibodies are the most specific for SLE, have a pathogenic relevance, and also correlate with disease activity [9,10]. Accordingly, the immunological background of SLE is very complex, and the main immunopathogenic mechanisms include efferocytosis defects (namely a reduced clearance of self-antigens, especially through complement factors, of which some patients have been shown to be deficient in), apoptosis defects (which also contribute to the loss of B-cell self-tolerance), and the inappropriate activation of type I interferon (which can sustain chronic inflammation and, thus, further compromises self-tolerance) [11,12,13,14]. Moreover, several innate cells have been implicated in the general dysregulation of the immunological environment, through the production of cytokines and/or co-stimulatory signals, which can support the production of autoantibodies and/or directly contribute to immune-mediated organ damage [15,16,17].

In this mini review, we aim to analyze the most relevant clinical studies providing information on the number and/or homeostasis and/or cytokine production of the DNT cell population in SLE patients, after summarizing the initial evidence from basic research (and, in detail, murine models), as regards a potential contribution of DNT cells in the immunopathogenesis of lupus.

## 2. DNT Cells and Lupus in Mice

Several murine models have been used to investigate the immunopathogenesis of SLE. The NZB/W F1 mouse is a F1 hybrid of the New Zealand Black (NZB) and New Zealand White (NZW) strains and develops a severe lupus-like phenotype, including a marked lymphoproliferation (lymphadenopathy, splenomegaly) in addition to elevated serum antinuclear autoantibodies (ANA) and, more specifically, anti-dsDNA IgG antibodies, which are associated with the development of immune complex-mediated glomerulonephritis resulting in kidney failure. Notably, these characteristics mainly appear in females, due to hormonal factors and, more specifically, estrogen levels [18,19].

The MRL/lpr mouse was derived from several crosses of inbred strains. Briefly, the MLR mouse genome is mainly derived from LG strain (75%) with a minor contribution from other strains (C3H: 12.1%; C57BL/6: 0.3%; and AKR: 12.6%). At the 12th generation of MRL mice inbreeding, a sub-strain with a spontaneous mutation in the *lpr* gene (which is located on chromosome 19 and encodes the FAS receptor) emerged; by cross-mating these mice, a *lpr*-mutated homozygous mouse strain (namely, MRL/lpr mouse) was obtained. This murine model displays a SLE-like phenotype with a shorter survival, compared to the NZB/W F1 strain and, notably, does not show any gender bias for the lupus-like phenotype [18,19,20].

In addition to these two main murine strains spontaneously developing an autoimmune disease, there are also “induced” lupus models, in which lupus-like manifestations are triggered by the exposure to specific environmental factors. Probably, the most well-known is the pristane-induced lupus model: here, the intraperitoneal injection of this isoprenoid molecule in BALB/c mice elicits the production of a variety of autoantibodies (including anti-DNA) along with the damage of several organs (such as the kidney, lungs, and joints) by immune-complex deposition. Notably, this mouse model shows a clear “interferon signature”, and the inhibition of IFN-I markedly reduces both autoantibody production and renal disease [18,21].

Since the MRL/lpr model is characterized by a strong lymphoproliferation sustained by the accumulation of DNT cells and displays a SLE-like phenotype with the development of nephritis and high titers of autoantibodies (including ANA, anti-dsDNA, and others), this experimental model has been largely used to investigate the potential role of DNT cells in general and, specifically, in lupus [21,22]. The expansion of DNT cells with the occurrence of massive lymphadenopathy in MRL/lpr mice has been noticed since the first description of this murine model [19]. However, the first mention of a specific role for this DNT cell population in lupus-related immunopathological aspects dates back to 1987 in a study by Datta et al., who investigated the production of anti-DNA antibodies and the role of T helper cells in murine models developing lupus nephritis. Briefly, they observed the presence of pathogenic anti-DNA cationic IgG in older mice developing lupus glomerulonephritis, whose production was supported by L3T4^+^ and Lyt-2^−^ T cells (corresponding to the CD4^+^CD8^−^ T cells) but also by a double-negative L3T4^−^Lyt-2^−^ T-cell population (namely CD4^−^CD8^−^ DNT cells); indeed, both these T-cell populations were expanded in the co-culture systems used to study the production of the aforementioned autoantibodies [23].

A comparable population was then described in humans shortly after. Indeed, in 1989, Shivakumar et al. described “the existence of an unusual Th population in the peripheral blood of humans that is CD4^−^CD8^−^ and TCRαβ^+^. These double-negative Th cells were markedly expanded in patients with the autoimmune disease SLE and, along with CD4+ Th cells, they induced production of the pathogenic variety of anti-DNA autoantibodies that are IgG in class and cationic in charge” [24].

Since then, several studies in MRL/lpr mice have investigated such a DNT cell-related lymphoproliferation and expansion, as regards its potential implication in lupus manifestations, especially nephritis. Some research showed that DNT cells can produce large amounts of IL-17 and other cytokines, can infiltrate the kidneys, and can support B cells in the production of autoantibodies, including pathogenic ones [25]. One study specifically showed that IL-17-deficient mice are protected against lupus; this finding was associated with a reduced frequency of DNT cells and, conversely, with the expansion of CD4^+^ regulatory T cells [26]. Further research in MRL/lpr mice proposed that the inhibition of DNT cells producing IL-17 could significantly suppress the development of lupus nephritis; indeed, MRL/lpr mice had been shown to have increased numbers of Th17 cells, which (upon IL-23 conditioning) could induce renal disease when they were transferred into RAG-1^−/−^ mice. Moreover, DNT cells were found to be largely represented among IL-17-expressing T cells that infiltrate nephritic kidneys [27,28,29,30,31].

Therefore, experiments in murine models suggested that some role may be effectively played by DNT cells in lupus and its complications, such as nephritis. However, these murine models of lupus are not completely consistent with their human SLE counterpart, of course, from both clinical and pathological points of view. Thus, an immunopathogenic mechanism emerging from mice cannot be automatically translated to humans, and experimental therapeutic approaches may have different outcomes across these two species. In this specific case, the protean clinical picture of SLE (and, probably, its underlying multifactorial etiopathogenesis) cannot be completely reproduced in the aforementioned experimental animal models [32]. Indeed, despite the significant contribution of basic research for the understanding of disease mechanisms, in order to answer the questions of whether DNT cells have a role in (human) SLE and, if so, ascertain its relevance in the disease pathogenesis, it is important and essential to directly investigate this rare T-cell population in clinical studies.

## 3. DNT Cells and Systemic Lupus Erythematosus

As already mentioned, after considering the initial evidence on the potential contribution of DNT cells to the production of pathogenic autoantibodies in the MRL/lpr murine model of lupus nephritis [23], Shivakumar et al. (1989) first investigated DNT cells in patients affected with SLE. They reported that DNT cells were markedly expanded in these patients and could contribute to the production of pathogenic anti-DNA IgG with cationic charge, along with conventional CD4^+^ T lymphocytes. In numerical terms, they also observed a statistically significant increase in DNT cell percentage in both active and inactive SLE patients (compared to controls); moreover, among patients, in the former group the number of DNT cells was significantly higher than in the latter one [24]. Several years later, Liu et al. (1998) also observed a greater percentage of DNT cells in SLE patients than in controls, but they found neither association with lupus nephritis nor correlation with disease activity or anti-DNA antibody titers [33].

Sieling et al. (2000) investigated the cytokine production of DNT cells in SLE patients. In addition to confirming an increased proportion of DNT cells, these authors showed that DNT cell lines derived from SLE patients could produce both IL-4 and IFN-γ and were also able to support IgG production by CD1c^+^ B cells, for which DNT-related IL-4 secretion was found to be important. Interestingly, DNT cells from healthy controls were also able to produce IFN-γ but not IL-4 [34]. However, the cytokine production pattern of DNT cells may be variable, as demonstrated by experiments with murine models in different pathological settings [2]. Indeed, Crispin et al. (2008) identified DNT cells as an additional and important source of IL-17 (in addition to IFN-γ), along with conventional CD4^+^ T lymphocytes. The demonstration that DNT cells represent a part of those IL-17-producing lymphocytes infiltrating the kidneys of SLE patients with nephritis further supported the hypothesis of a role for DNT cells in the immune dysregulation and/or organ damage observed in human SLE [35].

Eventually, Lai et al. published two studies (2012; and 2013) assessing some specific molecular aspects of T-cell dysregulation in SLE patients and, more specifically, the role of mitochondrial dysfunction in the activation and death pathways of these cells. In this bulk of experiments, these authors also reported some observations related to human DNT cells. In a randomized, double-blind, placebo-controlled trial assessing the safety and efficacy of N-acetylcysteine (which is a precursor of glutathione and has anti-oxidant properties that can improve the mitochondrial function) in SLE patients, they also observed an expansion of DNT cells compared to matched healthy controls [36]. In another study, they focused on the mammalian target of rapamycin (mTOR) to further study mitochondrial dysfunction in T cells: here, Lai et al. showed that mTOR activation can increase the production of IL-4 by DNT cells and their rate of necrosis, which may ultimately affect the balance of T regulatory cells and the production of pathogenic autoantibodies [37]. Eventually, the same research group described an increased production of IL-4 and IL-17 by DNT cells in a single-arm and open-label trial assessing the response to sirolimus in active SLE patients with resistance or intolerance to conventional therapeutic agents. Notably, 12-month sirolimus therapy also resulted in a reduction in the DNT cell circulating pool [38].

Previously, Dean et al. (2002) had already tried to investigate the intra-cellular content of IL-4 in DNT cells, and they observed a higher percentage of constitutively IL-4^+^ DNT cells in the peripheral blood of SLE patients than healthy controls (and even compared to some patients affected with rheumatoid arthritis) [39]. Notably, in this study and in another one (wherein DNT cell count was estimated after CD4/CD8/CD19/CD14-negative selection from peripheral blood mononuclear cells by using magnetic beads), these authors also reported an increased number of DNT cells in SLE patients. Even though they did not provide any quantitative information or figure related to the number of DNT cells, their research reported a greater frequency of IL-4^+^ DNT in SLE patients, in addition to other immunophenotypic differences in terms of activation markers [39,40].

The study by Tarbox et al. (2014) was the first one to mainly include pediatric SLE patients. These authors reported that 34.8% of SLE patients had increased DNT cell percentages in the peripheral blood, but this value (although increased) was not significantly different from that observed in other rheumatic children, such as those affected with juvenile idiopathic arthritis or mixed connective tissue disease; moreover, no healthy control group was included in the study [41]. Conversely, the large study (including 120 SLE patients between adults and children) by Wang et al. (2014) reported that SLE patients had more circulating DNT cells than patients with rheumatoid arthritis and healthy controls. These authors reported a positive and significant correlation with disease activity, in terms of SLEDAI. Notably, they also reported that the DNT numbers negatively correlated with their Fas expression, especially in active SLE patients [42].

The study by El Sayed et al. (2017) provided some more clues on DNT homeostasis and relevance in pediatric patients with SLE. They observed a significantly higher DNT cell percentage in the blood of SLE children with proliferative nephritis than in those with non-proliferative form, even though their number was comparable between patients with and without nephritis. Although no significant correlation was shown between DNT cell number and inflammatory parameters, such as ESR, or disease markers, like anti-dsDNA antibody titers or serum C3 levels, DNT cell percentage was shown to significantly and positively correlate with SLEDAI-2K score. Moreover, an increased percentage of DNT cells was observed more frequently in children with active SLE than among those in remission and, notably, none of their healthy controls showed such an increase of DNT cells. Finally, children with a new SLE diagnosis showed significantly more DNT cells than those with longstanding disease under treatment [43]. The study by Alexander et al. (2020) also included pediatric SLE patients as a minor part of their article, which was mainly focused on experimental findings from the MRL/lpr murine model. They showed an increased number of DNT cells in the kidneys of these children and also reported that 53% of them had an elevated number of DNT cells in the peripheral blood, which also correlated with kidney function (expressed as blood ureic nitrogen) [44].

Unlike most of the previous studies, Stratigou et al. (2017) did not observe any significant difference in DNT cells among active SLE patients, inactive SLE patients, and controls. However, their study aim was mainly the assessment of SLAM-family receptors expression on T lymphocytes, which was also measured in the DNT cell population. In this regard, their main DNT cell-related finding was that SLAMF6 expression on these cells could correlate with the response to B-cell depletion after rituximab [45].

A recent study by Li et al. (2020) provided some human data on DNT cells from SLE patients, along with a much more consistent part regarding experiments in mice, in order to study the interaction between marginal-zone macrophages and DNT cells. These authors reported a statistically significant increase in peripheral DNT cells in blood from SLE patients compared to healthy controls; additionally, they tested Ki67 expression on circulating T cells, which showed a statistically significant increase in the percentage of Ki67^+^DNT cells in SLE patients compared to healthy controls. Notably, they also reported the preferential usage of Vβ5 and Vβ8 by both CD8^+^ and DNT cells from SLE patients, unlike healthy donors. Thus, in addition to suggesting that DNT cells could undergo clonal expansion in a (self-)antigen-dependent manner, they considered that DNT cells might develop from self-antigen-stimulated CD8^+^ T cells in SLE patients [46].

All these studies are schematically summarized in Table 1. Overall, most studies seem to support an increased number of DNT cells in SLE patients. However, several aspects should let us carefully consider this observation. First of all, the majority of these investigations have a cross-sectional study design and, notably, most patients were not pharmacologically naïve and/or were sampled at different points during their disease course. Moreover, the range of the DNT cell increase in SLE patients is quite variable according to different studies, which could be due to the heterogeneity of several methodological aspects, including FACS equipment and gating strategy for DNT cells, in addition to the study population and sample timing. Therefore, even though the comparison with control patients allowed the researchers to observe a relative increase in DNT cell number in the peripheral blood of SLE patients, it is not possible to define this increase in absolute terms with these limited data.

## 4. Knowledge Gaps and Perspectives on Human DNT Cells

Lupus murine models supported a role of DNT cells in the production of immunomodulatory cytokines (including, but not only, IL-17) and in supporting B cells that can produce pathogenic autoantibodies, especially in the context of nephritis [23,24,25,26,27,28,29]. It is worth noticing that DNT cells have been highlighted as an important source of IL-17 in patients affected with Sjogren syndrome, where they were also increased in the peripheral blood and were shown to infiltrate the salivary glands [47,48]. Again, IL-17-producing DNT cell skin infiltration was described in patients affected with plaque-type psoriasis [49].

Unfortunately, human studies providing information on the homeostasis of DNT cell populations in SLE patients are very few so far; however, most of them showed that DNT cells are often increased in these patients, especially in phases of active disease [33,34,36,39,42,43,44,46]. Although no clear correlation with disease activity and/or inflammatory parameters has been established, the two most recent studies showed that DNT cell infiltration in the kidneys of SLE patients was increased and correlated with renal function (in terms of blood ureic nitrogen levels) [45] and displayed a more active/proliferative status according to their expression of Ki67 [46].

**Figure 1:**
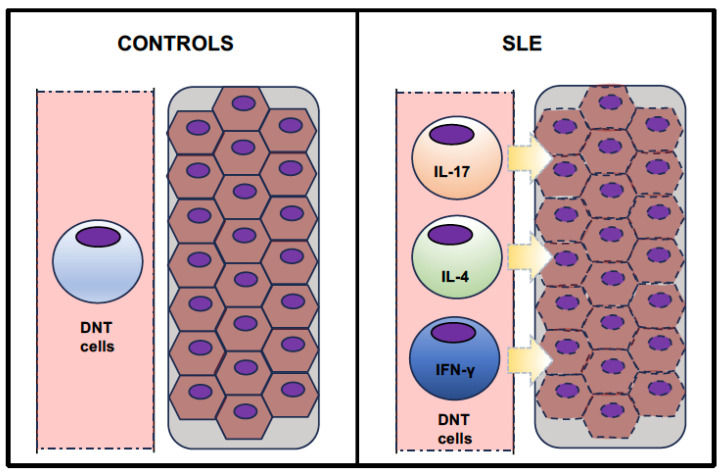
Graphical summary of the expansion and cytokine production of DNT cells in SLE patients.

As graphically represented in Figure 1, despite the small number, the available clinical studies overall observed an expansion of DNT cells in the peripheral blood of SLE patients. Moreover, some research also showed that DNT cells infiltrate the kidneys in SLE patients with nephritis, wherein these cells represent part of the lymphocyte pool (along with conventional T cells). Finally, DNT cells from SLE patients have been shown to be able to variably produce several cytokines, especially IL-17, IL-4, and IFN-γ.

Indeed, studies in murine experimental models highlighted the existence of several types of DNT cells according to their immunophenotypic characteristics and, perhaps, related functional aspects [1,4]. For instance, the “Th-like” phenotype of DNT cells has been proposed since these cells can secrete several cytokines, including IL-4, IL-17, IFN-γ, and TNF-α, which may regulate the immune response and/or the inflammation in several disease models, including those related to autoimmunity [4,50]. Moreover, “immunoregulatory” DNT (DNT_reg_) cells have been also implicated in immunological tolerance against alloreactive and autoreactive T cells, through both antigen-specific and non-antigen-specific pathways. These DNT_reg_ cells could exert their tolerogenic action towards both CD4^+^ and CD8^+^ conventional T cells, by inducing apoptosis through the Fas–FasL and/or perforin–granzyme pathways [4,51,52,53,54]. From a specific immunophenotypic point of view, some murine DNT cells were also shown to express those memory markers that usually allow one to discriminate between naïve and memory conventional T cells and, inside the latter group, between central and effector memory subpopulations [4,55]. Similar immunophenotypic aspects among DNT cells have been observed in recent human studies from different pathological settings [41,56,57,58,59].

Unfortunately, no clinical studies extensively assessed these immunophenotypic markers in SLE patients (except for the study by Anand et al.) [41], and this could be a relevant objective for future human studies, in addition to better defining the expression of cytokines produced by human DNT cells, in the context of SLE and other rheumatic diseases. Such investigations could highlight disease-related imbalances among specific DNT cell subsets in SLE patients (and, perhaps, in patients affected by different rheumatic disorders), which may provide new disease markers and, potentially, more personalized treatments, in addition to shedding light on the role of this specific T-cell population. Indeed, although the research efforts on DNT cells in SLE patients have not been very intense and have not increased over recent years, these cells still attract a lot of attention in several pathological settings beyond autoimmunity, such as in infections, organ transplants, graft-versus-host disease, and cancer [4,25,59,60,61,62,63]. Therefore, it may be worth performing further clinical research on the homeostasis and immunophenotypic aspects of DNT cells in patients with SLE and, perhaps, other rheumatic disorders. In fact, this review highlighted a knowledge gap that should be filled, since the role of DNT cells in autoimmune disorders (including SLE) could have been overlooked for several reasons (including the difficulties in studying rare cell populations in human patients). 

DNT cells expansion might not be just an unspecific epiphenomenon of autoimmunity. Our idea is that such an increase in DNT cells in SLE patients may have pathophysiological relevance in this disease. Indeed, a few studies included patients with other rheumatic disorders, in addition to the control group, as a term of comparison for SLE patients. Those two studies by Dean et al. and Anand et al. reported some quantitative and/or qualitative differences (in activation state or cytokine expression) in SLE compared to patients with rheumatoid arthritis, in addition to healthy controls [40,41]. Another study (by Tarbox et al.) observed a greater proportion of SLE children having increased DNT cell percentages (>2.5% of TCRαβ^+^CD3^+^ cells) compared to other rheumatic children (such as those affected with juvenile idiopathic arthritis), although this frequency was not significantly different [39]. The study by Wang et al. also included patients with rheumatoid arthritis: this research clearly showed a significant increase in DNT cell count in SLE patients compared to other rheumatic patients; moreover, these authors also observed that active SLE patients conserved such a significant difference against patients with rheumatoid arthritis, which was actually lost if only inactive SLE patients were used for this comparison [42].

Therefore, the presence of differences in DNT cells between SLE patients and other rheumatic patients might further support a specific immunopathological role of DNT cells in SLE, compared to the contrary hypothesis that these alterations could be an unspecific epiphenomenon of autoimmunity. Indeed, in our previous flow cytometry-based studies, we also confirmed another interesting peculiarity of immune cell homeostasis in SLE children, namely a reduction in basophils in their peripheral blood (compared to both controls and patients affected with juvenile idiopathic arthritis) [64,65], as previously shown mainly in adult SLE patients [66,67]. This additional example of peculiar alterations of specific immune cells (like basophils) compared to another rheumatic disorder led us to speculate about the immunopathological relevance of these changes, also considering the respective potential contribution of both basophils and DNT cells in the promotion of Th2-skewed immune response (also by producing IL-4) [68,69] and autoantibody production (by supporting B cells) [23,34], according to both general and specific evidence in mice. Moreover, further research investigating both these rare immune cells concomitantly in the same patients might reveal potential and functional connections. For instance, some research showed that murine CD8^+^ T cells that are activated in the presence of IL-4 can acquire a CD8^−^CD4^−^ immunophenotype [70], and IL-4 can promote the development of non-cytolytic T cells with low CD8 expression [71].

## 5. Conclusions

In summary, DNT cell homeostasis seems to be altered in patients with SLE, according to most of the available human studies, which included both adults and children, overall. In general, DNT cell percentage can be increased in SLE, especially in active patients; however, no clear correlation with disease activity and/or inflammatory parameters has been established. However, evidence regarding the role of DNT cells in SLE is very limited. Therefore, prospective and longitudinal clinical studies focused on the investigation of DNT cell populations should be planned, in order to further elucidate whether DNT cell expansion is an unspecific epiphenomenon of autoimmunity or, conversely, they directly contribute to SLE pathogenesis and complications; and, if so, to understand whether their number and/or immunophenotypic aspects may represent potential disease markers or even therapeutic targets.

## Figures and Tables

**Table 1:** Main clinical studies including information on DNT cells in patients with SLE.

Authors, Year, Country	Study Design	Primary Study Aim	SLE Pts. (*n*)	SLE pts. (Gender, Age)	SLE pts. Disease Duration	SLE Groups	SLE Groups (n, Age)	SLE Groups’ Disease Duration	Controls [n; Gender; Age]	DNT Cells Immuno-phenotype	DNT Cells [% CD3+]	Flow Cytometry Equipment	Therapy	DNT Cell-Related Findings
**Shivakumar** et al., **1989**, **USA** [24]	Prospective Cross- sectional	- To investigate the production of cationic anti-DNA IgG in lupus nephritis and cellular mechanisms regulating this process.	20	*M:F* = 3:18 *Age* n/a	n/a	- Active (with nephritis) - Inactive	n = 12 *range* 22–34 yrs. n = 8 *range* 35–55 yrs.	*range* 0.5–12 yrs. *range* 8–12 yrs.	n = 8 *M:F* = 3:5 *range* 20–30 yrs.	CD3+ CD4− CD8− TCRαβ+	*Mean ± SD* **Active**: 2.7 ± 0.80 **Inactive**: 0.9 ± 0.06 **Controls**: 0.27 ± 0.09 I vs. C *[p < 0.001]* A vs. C *[p< 0.001]* A vs. I *[p< 0.001]*	FACScan	n/a	- DNT cells were markedly expanded in SLE patients and, along with CD4+T cells, supported the production of pathogenic anti-DNA IgG with cationic charge.
**Liu** et al., **1998**, **Taiwan** [33]	Prospective Cross- sectional	- To investigate DNT cells in the peripheral blood mononuclear cells of SLE patients.	47	*M:F* = 4:43 *Mean* *(range)* 30 yrs. (12.0–58.0)	n/a	- Active (with nephritis) -Inactive	n = 26 n = 21	n/a n/a	n = 44 *M:F* = 3:41 “Similar age”	CD3+ CD4− CD8− TCRαβ+	*Mean ± SD* **SLE**: 1.14 ± 0.88 **Controls**: 0.88 ± 0.54	FACSsort	- “Majority of patients were taking variable doses of steroids”. - Cytotoxic drugs (n = 21)	- Increased number of DNT cells was found in SLE patients, but neither association with lupus nephritis nor correlation with disease activity and anti-DNA titers was observed.
**Sieling** et al., **2000**, **USA** [34]	Prospective Cross- sectional	- To investigate DNT cells and mechanisms leading to IgG autoantibody production in SLE.	20	*M:F* = 2:18 *Mean* *(range)* 39.1 yrs. (13–68)	*Mean* 8.7 yrs (0.5–25)	-	-	-	Yes (n = n/a) *F* = 57% *Mean* 32 yrs.	CD3+ CD4− CD8− TCRαβ+	*Mean ± SD* **SLE**: 3.0 ± 0.4 **Matched** **donors**: 0.6 ± 0.1 **Unmatched donors** 1.0 ± 0.2 *[p* <0.005]	n/a	Prednisone (0–40 mg/die) Cytotoxic drugs (n = 6)	- DNT cells from SLE patients produced both IL-4 and IFN-γ and supported CD1c1+ B cells to produce IgG antibodies.
**Dean** et al., **2002**, **UK** [39]	Prospective Cross- sectional	- To assess the percentage of IL-4^+^ DNT cells from patients with SLE and compare them with conventional T lymphocytes.	50	*M:F = 1:49* *Mean* *(range)* *37.2 yrs.* *(17–66)*	n/a	Variable disease activity	-	-	n = 16 *M:F* = 3:13 *Mean* *(range)* 36.1 yrs. (21–57)	CD3+ CD4− CD8− TCRαβ+	n/a ^$^	FACScan	- SLE patients were on steroid and/or Immuno- suppressive drugs, but no detailed information.	- IL-4^+^DNT cells were more frequent in peripheral blood of patients with SLE than healthy controls.
**Crispin** et al., **2008**, **USA** [35]	Prospective Cross- sectional	- To investigate DNT cells and their cytokine production in patients with SLE.	24	*M:F* = 0:24 *Mean* *(range)* 40.2 yrs. (25–57)	n/a	Variable disease activity	-	-	n = 16 n/a	CD3+ CD4− CD8− TCRαβ+	n/a ^$^	FACSAria	“Prednisone was discontinued at least 24 h before venipuncture”.	- DNT cells from SLE patients can produce IL-17 and IFN-γ. In detail, IL-17 producing cells and DNT cells are present in kidney biopsies of SLE patients.
**Lai** et al., **2012**, **USA** [36]	Prospective, Controlled, Double-blind trial	- To assess the safety, tolerance, and efficacy of the GSH precursor NAC and its related immunobiologcal impact.	36	*M:F* = 2:34 *Mean ± SEM* *(range)* 44.6 ± 1.8 yrs. (25–64)	n/a	Inactive (or stable disease)	-	-	n = 42 *M:F* = 3:39 *Mean ± SEM* *(range)* 44.4 ± 1.7 yrs. (22–63)	CD3+ CD4− CD8−	*Mean ± SD* **Baseline**: 6.2 ± 0.5 **After** **3-mo** **NAC**: 5.3 ± 0.5 *[p = 0.043]*	n/a	n/a	- “The mean±SEM 1.35± 0.12-fold DNT cells in patients with SLE compared to matched healthy controls (p = 0.008) was eliminated by NAC treatment, which also increased the mitochondrial hyper-polarization, mass, and apoptosis of DNT cells in SLE patients”.
**Lai** et al., **2013**, **USA** [37]	Prospective Longitudinal	- To assess the mitochondrial dysfunction and mTOR activation in peripheral blood mononuclear cells from SLE patients.	59	*M:F* = 3:56 *Mean ± SEM* *(range)* 43.1 ± 1.6 yrs. (20–65)	n/a	-	-	-	n = 54 *M:F* = 7:47 *Mean ± SEM* *(range)* 39.1 ± 1.8 yrs. (20–62)	CD3+ CD4− CD8−	n/a ^$^	n/a	n/a	- mTOR activation increases the production of IL-4 and necrosis of CD3+/CD42/ CD82 DNT cells.
**Tarbox** et al., **2014**, **USA** [41]	Prospective Cross- sectional	- To assess DNT cells in several pediatric autoimmune diseases, including SLE.	23	*M:F* = 5:18 *Mean ± SD* *(range)* 13 ± 5 yrs. (2–25)	n/a	-	-	-	n = 28 *M:F* = 7:21 *Mean ± SD* *(range)* 17 ± 5 yrs. (7–25)	CD3+ CD56− CD4− CD8− TCRαβ+ TCRγδ−	*Mean ± SD (range)* **SLE**: 2.2 ± 0.9 (0.4–4.5)	n/a	- No cytotoxic drugs (n = 19) - Cytotoxic drugs (n = 17) - Steroids only (n = 3) - Steroids + cytotoxic drug (n = 15)	- A portion (34.8%, slightly higher than other rheumatic disease, but not significantly) of SLE patients showed increased number of DNT cells. In general, patients with increased DNT cell percentages showed increased CD45RA expression.
**Wang** et al., **2014**, **China** [42]	Prospective Cross- sectional	- To assess DNT cells, their Fas expression, and intracellular cytokine levels in SLE patients.	120	*M:F* = 9:111 *Mean* ± SEM *(range)* 29.6 ± 1.1 yrs. (9–63)	n/a	- Active - Inactive	n = 82 n = 38	n/a n/a	n = 43 *M:F* = 3:40 *Mean ± SEM* *(range)* 30.6 ± 1.4 yrs. (7–25)	CD3+ CD4− CD8− TCRαβ+	*Mean ± SEM* **SLE**: 2.32 ± 0.12 **Active**: 2.68 ± 0.16 **Inactive**: 1.55 ± 0.11 **Control**: 1.03 ± 0.09 I vs. C *[p < 0.001]* A vs. C *[p < 0.001]* A vs. I *[p < 0.001]*	FACS Calibur	n/a	- DNT cells are increased in SLE patients and their value positively correlated with disease activity. - Abnormal Fas expression was observed in DNT cells.
**El Sayed** et al., **2017**, **Egypt** [43]	Prospective Longitudinal	- To assess peripheral DNT cells in pediatric SLE and their potential correlation with disease activity and different organ damage.	21	M:F = 0:21 *Mean ± SD* *(range)* 13 ± 2 yrs. (10–17)	n/a	- new diagnosis (active) - previous diagnosis (active)	n = 12 n = 9	0 yrs. (diagnosis) *range* 0.5–3 yrs.	n = 20 *M:F* = 0:20 *Mean ± SD* *(range)* 14 ± 2 yrs. [11,12,13,14,15,16,17]	CD3+ CD4− CD8− TCRαβ+	*Median (IQR)* **Disease** **activity**: 3.7 (3.0–5.7) **Disease** **remission**: 1.4 (1.2–1.8) **Controls**: 1.0 (0.5–1.4) **Active** **New SLE**: 5.0 (3.7–5.9) **Active Old SLE**: 2.8 (1.7–3.4)	Epics XLTM Navios	- “All patients received corticosteroid treatment during the period of follow-up” - CPM (n = 7) - MMF (n = 7) - Rituximab (n = 3)	- DNT cell percentage was significantly higher in proliferative nephritis than in non-proliferative nephritis but was comparable between patients with and without nephritis. - Active patients had more frequent DNT cell increase than those in remission. - DNT cell percentages showed a significant and positive correlation with SLEDAI-2K score and were higher in newly diagnosed SLE patients.
**Stratigou** et al., **2017**, **United Kingdom** [45]	Prospective Longitudinal	- To investigate the expression of SLAM-family receptors on T lymphocytes, including DNT cells, from SLE patients with different disease activity.	30	*M:F* = n/a *Median* 34.5 yrs.	*Median (range)* 8 yrs. (0–35)	-Active (with nephritis) -Inactive	n = 19 n = 11	n/a n/a	n = 20 M:F = 4:16 *Median* *(range)* 34 yrs. (24–54)	CD3+ CD4− CD8−	*Mean ± SEM* **Active**: 5.75 ± 3.43 **Inactive**: 3.68 ± 1.77 **Control**: 5.25 ± 3.34 I vs. C *[p = ns]* A vs. C *[p = ns]* A vs. I *[p = ns]*	FACSVerse	MMF (n = 16) HCQ (n = 23) AZA (n = 6) Pred (n = 14) None (n = 1)	- The frequency of DNT cells expressing SLAMF2/4/7 receptors was markedly altered in SLE patients, but these differences did not correlate with disease activity. - SLAMF6 expression on DNT cells could correlate with the response to B-cell depletion after rituximab.
**Lai** et al., **2018**, **USA** [38]	Prospective, Single-arm, Open-label, Phase 1/2 trial	- To assess sirolimus in active SLE patients that were intolerant or resistant to conventional drugs.	40	M:F = 2:38 *Mean ± SD* *(range)* 45.4 ± 14.3 yrs. (18–71)	n/a	-	-	-	43 *Mean ± SD* *(range)* 45.3 ± 12.7 yrs. Matched for Gender and Ethnicity	CD3+ CD4− CD8−	n/a ^$^	n/a	n/a	- Increased production of IL-4 and-IL-17 by CD4+ T cells and DNT cells at baseline, which was reduced after 12 months of treatment with sirolimus. IFN-γ production increased during sirolimus treatment in both CD4+ and DNT cells. Mean mitochondrial mass in DNT cells was higher in patients than in controls at baseline, and there was a decrease trend during sirolimus treatment.
**Alexander** et al., **2020**, **USA** [44]	Prospective Cross-sectional	- To investigate the role of DNT cells in SLE and their potential impact on kidney disease.	50	M:F = n/a *Range* 7–15 yrs.	n/a	-	-	-	Yes n/a	CD3+ CD4− CD8−	*Mean ± SD* **SLE**: 10.0 ± 6.1 **Controls**: 6.5 ± 1.0	LSRII Contessa	n/a	- DNT cells were increased in kidneys of active SLE patients and correlated with kidney function, in terms of BUN levels.
**Li** et al., **2020**, **USA** [46]	Prospective Cross-sectional	- to study the interaction between marginal-zone macrophages and DNT cells.	n/a	n/a	n/a	-	-	-	Yes n/a	CD3+ CD4− CD8− CD56- TCRαβ+	Done ^$^	n/a	n/a	- DNT cells were significantly increased in blood from SLE patients compared with healthy controls. - Moreover, Ki67^+^ DNT cells were also more represented in SLE patients (both in blood and kidney biopsies).

Abbreviations: M: male; F: female; n: number; n/a: information not available; yrs.: years; mo: months; SD: standard deviation; SEM: standard error mean; SLE: systemic lupus erythematosus; DNT: double-negative T cells; CD: cluster of differentiation; CD3: cluster of differentiation 3; CD4: cluster of differentiation 4; CD8: cluster of differentiation 8; TCRαβ: T-cell receptor alpha beta; CD56: cluster of differentiation 56; TCRγδ: T-cell receptor gamma delta; IL-4: interleukin-4; IFN-γ: interferon-γ; IL-17: interleukin-17; NAC: N-acetylcysteine; GSH: glutathione; mTOR: mammalian target of rapamycin; SLEDAI-2K: Systemic Lupus Erythematosus Disease Activity Index 2000; SLAMF2/4/7/6: signaling lymphocytic activation molecule family members 2, 4, 7, and 6; BUN: blood urea nitrogen, MMF: mycophenolate mofetil; HCQ: hydroxychloroquine; AZA: azathioprine; Pred: prednisone. ^$^: DNT cells were measured, but the DNT cell results are presented only in figure and numerical values are not shown in any table (refer to the last columns of the present table for the main qualitative findings in this regard).

## Data Availability

Not applicable.
